# The Role of Religions in the COVID-19 Pandemic: A Narrative Review

**DOI:** 10.3390/ijerph20031691

**Published:** 2023-01-17

**Authors:** Leuconoe Grazia Sisti, Danilo Buonsenso, Umberto Moscato, Gianfranco Costanzo, Walter Malorni

**Affiliations:** 1 Center for Global Health Research and Studies, Università Cattolica del Sacro Cuore, 00168 Rome, Italy; 2 National Institute for Health, Migration and Poverty (INMP), 00153 Rome, Italy; 3 Department of Woman and Child Health and Public Health, Fondazione Policlinico Universitario A. Gemelli IRCCS, 00168 Rome, Italy; 4 Dipartimento di Scienze Biotecnologiche di Base, Cliniche Intensivologiche e Perioperatorie, Università Cattolica del Sacro Cuore, 00168 Rome, Italy

**Keywords:** COVID-19, religion, health determinants

## Abstract

Culture, religion and health are closely intertwined, profoundly affecting people’s attitudes and behaviors as well as their conception and experience of illness and disease. In order to analyze the impact of religion in the current COVID-19 pandemic, we performed a literature review investigating both the scientific and grey literature on the topic. COVID-19 outbreaks reported in pilgrimages and religious ceremonies around the world—especially in the first wave of the pandemic wave—and the role played by religion in conveying culturally sensitive information about COVID-19 are some of the evidence we reviewed. Our research highlights how religions have represented, on the one hand, a risk for the spread of the virus and, on the other, a precious opportunity to engage people, and in particular minorities, in fighting the pandemic. To overcome this pandemic and to be prepared for similar ones in the future, scientists, politicians and health professionals should acknowledge the role that culture and religion play in people’s lives and how it can assist in tackling complex health challenges.

## 1. Introduction

The COVID-19 pandemic has disrupted the global economic, health and social scenario. Such a challenge cannot be tackled without considering the role that the cultural and social dimensions play in influencing pandemic resilience, compliance with public health measures, and the global commitment needed to overcome this unprecedented crisis.

Among cultural dimensions, religion undoubtedly covers a prominent role. The concepts of health and disease are deeply rooted in religious beliefs and people’s beliefs and behaviors may positively or negatively influence both individual and public health. It is the case of a diverse propensity to individual lifestyle habits, namely smoking, alcohol consumption, physical activity, dietary patterns [1,2], anti-conservative behaviors [3], or the risk of infection spreading due to religious and traditional rituals (e.g., ritualistic bathing and mortuary rituals) [4].

Moreover, historically, infectious diseases have assumed relevant religious connotations, for instance, those of divine punishment in leprosy and Ebola outbreaks [5,6]. Especially during the first pandemic wave, religious gatherings went under the spotlight as a relevant source of the virus spread [7]. Several worship ceremonies were canceled [8] or offered through livestream by churches, synagogues, mosques, and temples [9]. However, some worship ministers and religious groups ignored the restrictions on physical distancing, claiming exemptions for faith ceremonies [10], and questioned government guidance on SARS-CoV-2 prevention measures. Some religious houses also provided the faithful with disinfection supplies, personal protective equipment and free COVID-19 testing [11] or offered themselves as vaccination sites [12,13]. Nevertheless, some faith communities embraced conspiracy theories serving as promoters of COVID-19 misinformation [14].

Literature has also flourished on the role of religion, and more widely of spirituality, in coping strategies decreasing stress and promoting psychological well-being during the pandemic period [15,16]. The faithful’s concerns in the shift from embodied to disembodied religious practices impacting not only the religious rituals per se but also the social networks entrenched in religious meetings have been pointed out [17,18].

In this scenario, we aimed to capture relevant insights about the role held by religions in the COVID-19 pandemic, focusing on how religious habits and leaders have interplayed with the SARS-CoV-2 infection and its spread from the beginning of the pandemic to late 2021.

## 2. Materials and Methods

We performed a narrative review investigating both scientific and grey literature published on the topic from March 2020 to the 30th of September 2021. A search string was built using the keywords “COVID-19”, “SARS-CoV-2”, “Religion”, “religious confession”, “faith” and synonyms and variants. PubMed, Web of Science and Google Scholar were investigated and a hand-search on Google was also performed. Two researchers independently assessed all the records retrieved for their relevance to the research topic based on the abstract and the full text in two different consequential stages. Inclusion criteria were primary studies, short communications, perspectives articles and newspaper articles whose content dealt with the interplay between religion and COVID-19. Only literature published in English was considered for inclusion. Discrepancies in the selection process were solved through a consensus discussion among the two researchers.

## 3. Results

Sixty- one scientific articles, grey literature reports and newspaper articles were deemed relevant and narratively summarized according to the topics that emerged.

Topics identified were (1) religious pilgrimages and rituals worldwide being relevant to COVID-19 outbreaks, especially in the first pandemic wave (2) difficulties to engage the Closed Religious Communities (e.g., Haredi, Amish, etc.) in which community way of life, restrictions in using media and resistance to comply with preventive measures were identified as a significant COVID-19 risk (3) COVID-19 unofficial treatments (4) vaccine hesitancy also supported by concerns about the religious acceptability of vaccine composition or a firm interpretation of the Ramadan fasting (5) fuel of religious discrimination (6) religious communities and leaders strongly trusted in conveying COVID-19 information (7) religions playing a crucial role in coping stress and promoting mental (but also physical) well-being during the pandemic.

Literature review findings are presented and discussed here by categorizing them as risks and opportunities. The key characteristics of scientific articles and reports contributing to the different topics are reported in Table 1.

### 3.1. COVID-19 and Religion: Risks

#### 3.1.1. Religious Events and COVID-19 Spread

Religious events are traditionally characterized by large numbers of people meeting in dedicated places to pray or meditate, often for several days. Some rituals include also physical contact, such as shaking hands in the “sign of peace” in Catholic churches. Thus, such events can pose a relevant risk for the spread of respiratory viruses—including SARS-CoV-2—within the community. Previous respiratory disease outbreaks have shown that this risk is real. During the 2009 H1N1 influenza A and the Middle East respiratory syndrome coronavirus (MERS-CoV) outbreaks, face-mask use was low among Muslim pilgrims and, interestingly, the outbreaks coincided with the Hajj pilgrimage [19].

COVID-19 outbreaks associated with religious events were confirmed early in the pandemic, as early as February 2020 and the risk of large-scale religious events being pandemic triggers was also highlighted in *The Lancet* [19].

In Albany (Georgia, USA), the SARS-CoV-2 virus infected more than 100 people who went to a funeral in February 2020, fueling an outbreak in the surrounding rural county [20]. In Arkansas, a pastor infected more than 30 attendees in a religious ceremony, leading to three related deaths and the infection of 26 other people, one of whom died [20,21]. In Saudi Arabia, returning Saudi pilgrims visiting pilgrimage sites in Iran and Iraq were suspected to be the initial source of the spreading of SARS-CoV-2 in the community [22]. Luckily, for the first time in the eight decades of the history of Muslim pilgrimage, as soon as the pandemic began to worsen, the Kingdom of Saudi Arabia, on 27 February 2020, placed restrictions on the inbound Umrah pilgrimage [23]. Conversely, Pakistan did not stop religious travelers at first. On 24 March 2020, Pakistan reported 990 cases, sixty percent of which being pilgrims returning from Iran [24]. The same happened in Iran, where several cases within the country and a dozen in neighboring countries were linked to large pilgrimage events [25,26]. In Greece, 48 out of 53 (90.6%) pilgrims who visited Jerusalem on 19 February 2020, tested positive [27]. In Guangzhou (China), SARS-CoV-2 infection was diagnosed in six passengers returning from a pilgrimage in Pakistan. These pilgrims had spent the previous weeks in close contact with thousands of pilgrims gathered in a masjid, without wearing facemasks, thus potentially infecting a large number of people [28]. In Malaysia, in early March 2020, about 16,000–19,000 people of different nationalities attended the Sri Petaling gathering organized by a Muslim missionary movement and held in Kuala Lumpur [29,30]. One thousand seven hundred people tested positive and, later, 35% of new COVID-19 cases reported in the country were linked to this gathering [30]. This apparently acted as the source of infection for the next two gatherings in Pakistan and India as several infected Malaysians attended those meetings [31]. Moreover, in late February 2020, another COVID-19 cluster in Malaysia originated from a Christian leadership seminar in Kuching, Sarawak (a Malaysian state in Borneo). The event has been identified as the source of 117 of the 371 COVID-19 cases in Kuching [29]. In India, as of 4 April 2020, 1023 people related to this congregation had tested positive [32]. Another outbreak in the northwestern Indian state of Punjab was linked to a 70-year-old Sikh priest who, after returning from Italy and Germany, refused self-quarantine and attended several religious meetings, including a Sikh festival attracting 300,000 people daily [33]. In the Eastern Cape Province, South Africa, as of 7 May 2020, about 80% of the infections in the province resulted from three burial ceremonies in Port St. Johns, Port Elizabeth and Mthatha. In the Free State province (still in South Africa), a single religious event attended by three COVID-19-positive church leaders led to the infection of more than 80 people and the further tracing of 1600 people who may have been exposed to the virus [34].

In Italy, the high number of priests who have died of COVID-19 in the first pandemic waves (269 as of April 2021) [80] and the resulting shortage of hospital chaplains, led to nurses and doctors being appointed to give the blessing [81].

Especially in the first phases of the pandemic, COVID-19 infection rates among the ultra-orthodox Jews of Israel have been reported to be significantly high, considering the size of this group population in Israel [82]. Even if large families and crowded living conditions have been called upon for explaining this finding, participation in daily communal religious prayers (and the Jewish holiday of Purim on 9 March 2020) may also have contributed [35]. In London, about a thousand devotees of the International Society for Krishna Consciousness attended a funeral in its temple on 21 March 2020. Twenty-one of them tested positive and five died [36]. A similar outbreak occurred in Italy, in the Molise Region, after a Roma funeral [37].

A further example of the importance—for health—of a full understanding of religious and cultural contexts is offered by the ultra-orthodox community in the US who, due to cultural and social rules preventing them from using technological devices and media, was not promptly updated on the severity of the first pandemic wave of COVID-19 [38]. Similar considerations have been made for other closed religious communities (CRCs), such as the Amish/Mennonites, for whom technological restrictions, regular face-to-face worship rituals, and resistance to preventive measures (including vaccination) have raised concern about COVID-19 spread [39].

#### 3.1.2. COVID-19 Unofficial Treatments and Vaccine Hesitancy

Another potential risk linked to religious beliefs lies in the resistance to adopting COVID-19 preventive measures (including vaccination) due to the persuasion that God’s protection is the only effective resource and in the use of unofficial treatments tied to religious beliefs. About the latter, Lebanese Christians have been described as drinking a mixture of water and sacred soil found at the grave of Maronite monk Mar Charbel (Mount Lebanon); Hindu groups have been reported hosting cow-urine drinking parties to cure COVID-19 [40].

Focusing on vaccination, even before the spread of the pandemic, several studies have shown how religion can influence vaccine hesitancy, intended as a delay in accepting or refusing vaccines despite the availability of vaccination services [83]. A case study on measles and rubella vaccine hesitancy in Zimbabwe highlighted how religious teachings that emphasize prayers as alternatives to medicines, and the lack of privacy in a religiously controlled community can reinforce the hesitancy generated by poor knowledge of vaccine safety and effectiveness among members of the Apostolic Church [84]. Regarding COVID-19 vaccination campaigns, a national survey covering 638 Arab Americans—more than half foreign-born—between May 2020 and September 2020 highlighted that only 56.7% of respondents reported an intention to be vaccinated and that 7.5% stated being frankly unlikely to receive a vaccine. Of those, 85.4% self-reported a moderate to high religiosity and women were five times more unlikely to receive the vaccine than men, suggesting that relying on religion as a coping mechanism in facing the COVID-19 pandemic, coupled with possible misinformation about COVID-19 vaccines, informally spread throughout religious communities and posed additional barriers in vaccine literacy among Arab American women [41]. In the US, the “Religion and the Vaccine Survey”, conducted in March 2021 underlined that Protestants (Black, Hispanic, White evangelical and “other Protestant of color”) and Mormons were the least vaccine-receptive religious groups (share of accepters equal/less than 50%). Among these, White evangelical Protestants reported the higher share of vaccine refusers at 26%, whereas Hispanic Protestants were the most vaccine-hesitant (42%). An increase in acceptance has been observed in June 2021 [42].

Conspiracy narratives against COVID-19 vaccines tied to religious beliefs have been highlighted in Pakistan [43], but unscientific theories and COVID-19 vaccination misinformation promoted by religious leaders appeared to spare no religious belief, according to Galang [44].

Moreover, concerns about the acceptability of vaccine composition and manufacturing processes, such as porcine gelatin for Muslims and Hindus or cell lines from aborted fetuses for Christian communities, have also been expressed [45].

In addition, a firm interpretation of the practice of fasting during Ramadan, intended as “refraining from anything entering the body cavities,” has been feared to promote some reluctance to receive vaccinations during the holy month [46]. Attention has also been paid to the role of fasting in influencing the severity of SARS-CoV-2 infection, highlighting the need for future studies to better address the topic [73,74,75].

Furthermore, the pandemic may also fuel religious discrimination [47], in particular towards Muslims and Jews [48]. Beliefs that Jews developed the virus to later gain credit and profit from the vaccine they would develop or that the pandemic is a punishment for Jewish denial of Jesus are classic examples of observed anti-Semitic attitudes [48].

### 3.2. COVID-19 and Religions: Opportunities

Religious meetings represent meaningful moments for believers and may be particularly important for minorities. For example, Black churches have a well-known role in promoting spiritual renewal and mental resilience and in addressing racism, especially for older African Americans [59]. A proactive engagement of religious communities and leaders may ensure both significant relief during pandemic times and proper and trusted communication on how to appropriately deal with the pandemic, also considering the increase and the strengthening in religiosity that some studies have detectedduring the pandemic [60,61].

In this regard, proactive activities have been developed in some contexts. In São Paulo, a spiritual hotline was developed in May 2020, and, during the first two weeks, 108 appointments were requested, and calls were made from Brazilian states and from Portugal [62], highlighting the resonance of the project. In the Philippines, Catholic congregations organized online ceremonies providing online counseling and guidance, and distributed free protective equipment and food to the poor and homeless people [63]. Similar support services have also been set up in Detroit [49].

An online survey conducted in March 2020 in the first quarantined community in the US, a Modern Orthodox Jewish community, showed that community organizations were more trusted than any other source of COVID-19-related information by offering concrete support, such as food delivery, social support, virtual religious services, and dissemination of COVID-19-related information [50]. Religion can be used in disseminating precautionary measures and evidence-based practices against COVID-19 [51,52], as we have already learned in defeating the Ebola epidemic in West Africa [85].

Moreover, important religious ceremonies, such as the Jewish holiday of Pesach (or Passover), Ramadan, Easter, and funerals, were organized through online platforms [76,77], underlining how religion was able to adapt to the pandemic and reinforcing the relevance of complying with public health measures in the faithful. However, this shift has not been painless. The literature highlights that the forced digitization of religious worship disrupted religious habits and practices requiring the faithful to accept not physically participating in fundamental liturgical rituals, such as the Eucharist, the passing of the peace, the burning of incense for the Christians [18,78], and the holy chants. Besides the missing of physical participation in rituals, the faithful also expressed how the impossibility to meet in religious rituals (e.g., group prayers, pilgrimages) affected their social networks, often deeply rooted in religious belonging, being only partially relieved by online worship and meetings [18]. This is even more crucial within the context of religious and ethnic minorities, for which physical participation in rituals and meetings maintains and provides a transnational source of identification, constituting an essential factor in their social network [79]. The value of physical participation also emerges in a survey performed in the United Kingdom (UK), soon after the easing of restrictive measures (July 2020). The survey, covering 939 participants of different religious backgrounds, reported on the global compliance of worship places with social distancing and the use of face masks with a general acceptance of this latter. Face masks, even if perceived as uncomfortable and reducing the singing and chanting volume, were globally tolerated, since this meant resuming the singing and chanting during communal worship [53].

Furthermore, the role of religious coping—intended as the use of “cognitive or behavioral techniques, in the face of stressful life events, which arise out of one’s religion or spirituality” [86]—in easing COVID-19 anxiety and supporting psychological well-being during the pandemic has strongly come to the fore. During the early months of the pandemic, Google searches for prayer relative to all major religions collected for 107 countries rose by 30%, reaching the highest level ever recorded and remaining 10% higher than previously throughout the entire 2020 [61]. According to the author, this finding seems to indicate more than just the mere replacing of physical churchgoing with online worship but a global, increasing demand for religion as a means to cope with adversity. An online survey covering 1250 adults in Italy underlined that the participants, and in particular women, were perceived to have poorer mental health than in the pre-pandemic period, and that spirituality and religious practices play a protective role in psychological and mental health but also for physical health [64]. In a cross-sectional study on a sample of 419 American Orthodox Jews, negative religious coping and mistrust in God have resulted to correlate strongly with higher levels of COVID-19-related negative impacts in different areas of life (e.g., sleep, diet, family, relationships, enjoying life) versus a global and better resilience of individuals with intrinsic religiosity and positive religious coping [65]. In a sample of 970 Americans between 20 and 79 years of age, negative religious coping resulted in the likelihood of being more associated with COVID-19 anxiety than positive religious coping [66]. In a survey performed on 543 residents of the United Arab Emirates, in the early stages of the pandemic, Muslims reported significantly higher levels of positive religious coping compared to their Christian counterparts, and in this group, positive religious coping was found to be inversely related to depressive symptoms and having a history of psychological disorders [67].

In this regard, particular attention is paid to the elderly, who are usually the most religious in communities [87]. Geriatric psychiatrists believe that faith may support older patients in relieving anxiety during the COVID-19 pandemic [68] and female older adults have been found to have higher levels of religious coping, and lower levels of death anxiety during the COVID-19 pandemic than male older adults [69]. Moreover, in older homebound adults, positive religious coping has been reported to be associated with a less suicide risk [70]. The positive impact of religion and spirituality on mental health has also been highlighted in a cross-sectional study on a sample of 200 Malaysian healthcare workers involved in the assistance of COVID-19 patients, in which positive coping was predictive of a reduction in anxiety and depression scores [71]. The lowering of psychological distress has also been reported in a more recent study on a sample of 549 caregivers (parents and other adults in childrearing roles) across Canada, United States, United Kingdom, and Australia [72]. Thus, besides the role of religions in favoring compliance with COVID-19 preventive measures, also its role in coping strategies should be further valorized by governments and institutions, as scientists and researchers advocate [30,88]. The World Health Organization is moving in this direction by recognizing the importance of chaplaincy interventions in supporting the healing process of religious patients [89].

With the launch of the COVID-19 vaccination campaigns, the positive role of religion in promoting adherence to COVID-19 vaccination and in elucidating moral issues that can cause vaccine skepticism in their faithful has come powerfully to the fore. The “Religion and the Vaccine Survey” (United States) underlined how faith-based approaches can be effective for hesitant and refusing groups with about 40% of vaccine-hesitant (44% in March 2021 and 38% in June 2021) and 14% (March 2021) and 19% (June 2021)of vaccine-resistant Americans who attend religious services at least a few times a year saying that faith-based approaches would make them more likely to vaccinate [42]. The South Dakota COVID-19 Impact Survey (SDSU Poll), performed in April 2021, highlighted as among people who had not received a vaccine, those spurred by a religious leader indicated nearly twice the likelihood of getting vaccinated than those invited by politicians or medical professionals [54].

The Vatican Congregation for the Doctrine of The Faith has reassured Catholics about the moral legitimacy of receiving COVID-19 vaccines that have used cell lines from aborted fetuses in their research and production process in case of a lack of alternatives [55]. The same note also emphasizes that “the morality of vaccination depends not only on the duty to protect one’s own health, but also on the duty to pursue the common good”, especially with regard to protecting the weakest and most exposed [55]. Some Catholic churches have proposed themselves as vaccination sites [56] and in August 2021, Pope Francis urged people to get vaccinated against COVID-19 [57]. Similarly, appeals to join COVID-19 campaigns and reassurance about the religious acceptance of vaccine composition [58] have come from different religious leaders. Global faith leaders also called for rejecting vaccine nationalisms and embracing a commitment to global vaccine equity [90]. 

## 4. Limitations of the Study and the Way Forward

Our study aimed to investigate and provide an overview of key topics concerning the interplay between religion and the COVID-19 pandemic. As we conducted a narrative review, we cannot rule out that all available evidence on the topic has been considered. Moreover, the timeframe of the research has been confined to September 2021, even if some relevant latest articles have been mentioned.

While taking into account the limitations stated, some considerations arise. First, most of the literature retrieved on the topic does not provide primary data or has not been subject to peer review processes encouraging more scientific and original research to better inform healthcare practitioners and evidence-based policy-making. Further, the screened literature clearly showed a preponderance of studies led in Asian countries and the US and a lack of studies set in European countries, prompting the scientific production attention on the topic in these countries. 

## 5. Conclusions

Our review findings plainly push for an acknowledgement of the role that religion has in facing complex health challenges and adopting a phenomenological, anthropological, and cultural approach in designing public health strategies. The knowledge of the different cultural and religious specificities and cooperation with religious leaders are crucial to ensure that all the different groups are included in health policies and engaged in health production and protection.

This can be achieved through the establishment of inter-religious and pluricultural collaborative relationships with all representatives of different religious denominations, committed to promoting communication channels providing accurate, accessible, and reliable information to members of religious and belief communities, as also highlighted by a joint Statement of the International Religious Freedom Alliance [91]. Health systems and health policy should become more sensitive to religious and cultural issues, for example, by training the health workforce and structuring cultural and religious-sensitive health pathways. In turn, religious organizations can act as intermediaries to reach out to communities that may have difficulties in accessing health services or are resistant to implementing evidence-based measures.

Such an approach is of the highest priority even in this pandemic phase, which is newly characterized by high virus circulation, especially in some countries, and where the persistence of unequal availability of COVID-19 vaccines continues. Furthermore, our review of the concerns held about vaccine hesitancy and vaccine refusal movements is of significant value when facing possible future pandemic challenges.

## Figures and Tables

**Table 1:** Main topics and related references resulting from the literature review.

Topic	Author (First Name)	Year	Type of Publication	Country	Main Findings
COVID-19 outbreaks related to religious gatherings	Ebrahim SH [19]	2020	Short communication	Saudi Arabia	Umrah pilgrimage to Saudi Arabia as a potential superspreading event.
	Aschwanden C [20]	2020	Newspaper article	United States	Outbreaks due to religious events reported in Georgia, Washington and Arkansas states (February–March 2020).
	James A [21]	2020	National bulletin	United States	Arkansas outbreak due to a religious event (March 2020): 38% of 92 attendees tested positive. Additional 26 cases identified through contact tracing,
	Memish ZA [22]	2020	Short communication	Saudi Arabia	Returning Saudi pilgrims from Iran and Iraq as early source of SARS-CoV-2 spread, contributing to 150,000 cases.
	Ebrahim SH [23]	2020	Short communication	Saudi Arabia	Suspension of the Umrah pilgrimage to mitigate the COVID-19 spread risk.
	Badshah SL [24]	2020	Short communication	Pakistan	60% of cases identified in Pakistan by 24 March 2020 were pilgrims who travelled to Iran.
	Quadri SA [25]	2020	Perspective	Iran; Malaysia; Pakistan; India; Israel; South Korea	COVID-19 outbreaks due to Muslim, Hindu, Christian, Jewish, Sikh religious gatherings
	Wright R [26]	2020	Newspaper article	Iran	Fatima Masumeh pilgrimage in Qom as a source of COVID-19 spread in neighbouring countries
	Pavli A [27]	2020	Short communication	Israel	A cluster (48 cases) of SARS-CoV-2 infection in Christian Greek pilgrims returning from Israel in late February 2020
	Gu Y [28]	2020	Editorial	China	Six cases of SARS-CoV-2 positivity recorded among the passengers of a flight from Pakistan (late March 2020). Cases had attended a pilgrimage at a masjid in that country.
	Che Mat NF [29]	2020	Short communication	Malaysia	35% of new COVID-19 cases recorded in Malaysia in early April 2020 have been linked to the Muslim Sri Petaling gathering
	Tan MM [30]	2021	Perspective	Malaysia	Diverse COVID-19 outbreaks linked to religious gatherings in Malaysia.
	Najib M [31]	2020	Newspaper article	India	About 40,000 people quarantined in Punjab following a coronavirus outbreak linked to a single Sikh priest.
	Daim N [32]	2020	Newspaper article	Malaysia	About 44% out of the 3483 COVID-19 cases reported in Malaysia in early April 2020 linked to the religious tabligh gathering in Sri Petaling.
	Kumar P [33]	2020	Newspaper article	India	About 30% of all confirmed COVID-19 cases in India linked to the Tablighi Jamaat religious gathering in Delhi. More than 22,000 have been quarantined or isolated. The government has declared the event as the largest of 14 coronavirus hotspots across the country (April 2020).
	Jaja IF [34]	2020	Correspondence	South Africa	80% of all infections in the Eastern Cape Province ascribed to burial ceremonies in Port St Johns, Port Elizabeth and Mthatha. Over 80% of cases reported in the Free State derived from a single religious event leading to the infection of over 80 persons and the further tracing of 1600 (March–April 2020).
	Zalcberg S [35]	2021	Original research (cross-sectional)	Israel	Sample: 25 participants (17 men, 8 women; age range 25–60 years old) from various Ashkenazi groups of ultra-Orthodox society who had tested positive for COVID-19 or had contact with a COVID-19 case. Period: March-September 2020 Main results: Participants perceived as causes for the high COVID-19 infection rate amongst the ultra-Orthodox population: (1) population and housing density; (2) community way of life, including frequent and collective religious practices and (3) disobedience of the COVID-19 preventive measures.
	Tandon L [36]	2020	Newspaper article	United Kingdom	The International Society for Krishna Consciousness (ISKCON community) in London has reported at least 21 confirmed cases and 5 deaths among those who attended a funeral at the ISKON temple on 12 March 2020, weeks before Prime Minister Boris Johnson imposed a lockdown. At least 1000 devotees had gathered for the funeral.
	Il Messaggero [37]	2020	Newspaper article	Italy	An outbreak in a Roma community in early May 2020 had been attributable to a funeral ceremony. 72 positive cases had been estimated
Difficulty to penetrate Closed Religious Communities	Dalsheim J [38]	2020	Newspaper article	United States	Internet access, television broadcasts and certain cellphone functions are generally limited in strictly observant ultra-Orthodox Jewish communities. This prevented some observants to be timely informed about the virus spreading in the early phases.
	Stein RE [39]	2021	Original research (Retrospective study)	United States	Population: Amish and Mennonites community in Ohio Methods: 2020 vs. 2015–2019 excess death calculation based on obituary information published in the major Amish/Mennonite newspaper. Main results: Amish/Mennonite excess death rates globally similar to the national trends. Excess death rate spiked with a 125% increase in November 2020 when many governmental restrictions relaxed and many of the Amish and Mennonite groups were engaging in face-to-face interactions. According to authors, the importance of face-to-face rituals among CRCs indicates the spread of COVID-19 could be especially problematic within these groups, particularly for those that restrict technology.
COVID-19 unofficial treatments	Iqbal Q [40]	2020	Correspondence	Pakistan	Drinking cow urine and hosting cow urine drinking parties in Hindu communities. Combining and consuming water and sacred soil found at the grave of Maronite monk Mar Charbel was reported among some Lebanese Christians.
Vaccine hesitancy	Abouhala S [41]	2022	Original research (Cross-sectional study)	United States	Sample: 638 Arab Americans
					Period: May–September 2020 Main results: 56.7% reported the intention to be COVID-19 vaccinated; 35.7% reported uncertainty, and 7.5% reported being unlikely. Women had higher odds of being uncertain (OR = 1.68; 95% CI: 1.10, 2.57) or being unlikely to receive the vaccine (OR = 5.00; 95% CI: 1.95, 12.83) than men.
	Public Religion Research Institute (PRRI) staff [42]	2021	Original research (Cross-sectional study)	United States	Protestants (Black, Hispanic, white evangelical and “other Protestants of color”) and Mormons resulted to be the least vaccine-receptive religious groups (share of accepters equal/less than 50%) in March 2021. Increase in acceptance has been observed in June 2021.
	Khan Y [43]	2020	Short communication	Pakistan	Threat of COVID-19 Vaccine Hesitancy in Pakistan. Anti- COVID-19 vaccine conspiracy narratives often tied to religious beliefs and spread by political leaders
	Galang JRF [44]	2021	Correspondence	n.a.	Anti-vaccine misinformation promoted by leaders of different religions. Homosexuality, control of the mind, conspiracy to “feed cow’s blood to Hindus”, manufacturing based on slaughtered fetuses, “mark of the devil” are some of the arguments used.
	Seale H [45]	2020	Newspaper article	Australia	Religious concerns over vaccine production methods and the importance to engage religious leaders to ensure they are equipped with accurate information about the potential COVID-19 vaccine, its development process and the rationale for its use.
	Ali S [46]	2021	Correspondence	n.a.	COVID-19 vaccine concerns during Ramadan fasts.
Fuel of religious discrimination	Sarkar S [47]	2020	Feature	India; Pakistan; Cambodia; South Korea	COVID-19 as a pretence for religious discrimination.
	United Nations Press Release Staff [48]	2020	Press release	n.a.	Increase in conspiracy-driven anti-Semitic hate speech.
Role of religious communities and leaders in COVID-19 information					
	Modell SM [49]	2020	Philosophical exploration	United States	Spiritual and material support promoted by churches.
	Weinberger-Litman SL [50]	2020	Original research	United States	Sample: 308 Modern Orthodox Jewish Results: Community organizations trusted more than institutional and media sources in COVID-19-related information.
			(Cross-sectional study)		
	Levin J [51]	2020	Commentary	n.a.	Individual clergy and congregations as sources of COVID-19 misinformation and disinformation.
	Galiatsatos P [52]	2020	Original research (Case study)	United States	Target population: Faith community leaders, representatives from religious communities, senior centers, hospitals and other health care centers, community service organizations, and the local government. Intervention: 12 Community conference calls to disseminate CO VID-19 information and provide mental support. Period: March–April 2020 Main results: Advance care planning, telemedicine, social isolation, mental health, meditation and other coping strategies among the topics discussed. Information received has been shared throughout the community. Additional community calls were requested with particular regard to mental health. Distribution of food and facemasks was also achieved at three congregations. The calls also served to identify and correct any potentially harmful misinformation circulating among the communities and to prepare religious leaders for the safe re-opening of religious services.
	Ho KMA [53]	2022	Original research (Cross-sectional study)	United Kingdom	Sample: 1063 participants from different religious backgrounds. Period: August–November: 2020 Sample: 939. Main results: 939 respondents (80.7% self-identified as Christians), of whom 78% find it acceptable to wear a face mask during worship. 97.3% stated their place of worship complied with government guidelines and 90.5% stated that it enforced face mask-wearing.
	Wiltse D [54]	2021	University press release (Cross-sectional and comparative cross-sectional study)	United States	South Dakota COVID-19 Impact Survey (SDSU Poll) Population: 3057 registered voters in South Dakota Period: 12–25 April 2021 Main results: Among participants who had not received a vaccine, those spurred by a religious leader indicated nearly twice the likelihood of getting vaccinated than those invited by politicians or medical professionals.
	Congregation for the Doctrine of The Faith [55]	2020	Congregation for the Doctrine of The Faith	n.a.	COVID-19 vaccines that have used cell lines from aborted fetuses in research and production process are morally legitimate in case of a lack of alternatives. Pharmaceutical companies and government health agencies are asked to be committed to producing, approving, distributing ethically acceptable vaccines that do not create conscience concerns and are accessible also to the poorest countries.
			Official Note		
	Lacsa JME [56]	2022	Correspondence	n.a.	Catholic Church supported government vaccination programmes by offering churches as vaccination sites. Moral acceptability of vaccines in line with the Official Note of the Congregation for the Doctrine of the Faith.
	Vatican News [57]	2021	Newspaper article	n.a.	Pope Francis urges people to get vaccinated against COVID-19 adding that “getting vaccinated is a simple yet profound way to care for one another, especially the most vulnerable”.
	Mirza Asad [58]	2021	Newspaper article	n.a.	Leaders of different religions (Islam, Orthodox Judaism) reassure about the religious acceptance of vaccine composition.
Role of religions in coping COVID-19 stress and promoting mental and physical well-being	DeSouza F [59]	2021	Perspective	United States	Physical closure of churches increased mental stress of the faithful. Black Churches promoted spiritual renewal alongside mental resiliency and coping against societal racism, especially for older African Americans.
	Gecewicz C [60]	2020	Research Center Report	United States	Pew Research Center’s American Trends Panel survey Sample: 10,139 US adults Period: 20 to 26 April 2020 Main findings: One-quarter of U.S. adults overall (24%) say their faith has become stronger because of the coronavirus pandemic,
	Bentzen JS [61]	2021	Original research (Observational study based on Google searches)	n.a.	During the early months of the pandemic, Google searches for prayer rose by 30%, reaching the highest level ever recorded. The rise was observed in all continents, at all levels of income, inequality, and insecurity, and for all types of religion, except Buddhism.
	Ribeiro MRC [62]	2020	Correspondence	Brazil;	Spiritual Hotline Project aimed to offer free spiritual and religious assistance and to make a referral, if needed. Time frame: 29 May–14 June 2020
			(including a case report)	Portugal	Results: 108 appointments requested and calls to the free telephone hotline made from 107 Brazilian states and 2 countries (Brazil and Portugal).
	Del Castillo FA [63]	2020	Correspondence (including a case report)	Philippines	Roman Catholic Church initiatives in the Philippines: online-based religious ceremonies; online counselling and pastoral guidance to increase coping; personal protective equipment provision; feeding support to the poor
	Coppola I [64]	2021	Original research (Cross-sectional study)	Italy	Sample: 1250 adults Period: February–May 2020. Main results: Participants perceived lower levels of spiritual well-being and mental health than the pre-pandemic situation and women perceived lower mental health than men. Spirituality and religious practices as protective factors for physical and mental health. Family as a protective factor for mental health.
	Pirutinsky S [65]	2020	Original research (Cross-sectional study)	United States	Sample: n = 419 American Orthodox Jews Period: March–April 2020 Main results: positive religious coping, intrinsic religiosity and trust in God strongly correlated with less stress and a more positive impact.
	DeRossett T [66]	2021	Original research (Cross-sectional study)	United States	Sample: 970 participants Period: 12–25 September 2020. Main Findings: negative religious coping positively associated with COVID-19 anxiety. Positive religious coping negatively, although weakly, associated with COVID-19 anxiety.
	Thomas J [67]	2020	Original research (Cross-sectional study)	United Arab Emirates	Sample: 543 Muslim and Christian residents of the United Arab Emirates (UAE) Period: 6–17 April 2020 Main results: Positive religious coping was inversely related to having a history of psychological disorders. Muslims reported significantly higher levels of positive religious coping compared to Christians.
	Koenig HG [68]	2020	Commentary	n.a.	Religion as a relevant resource for health and well-being in older adults. Geriatric psychiatrists can help religious elders make use of their faith to relieve anxiety during the COVID-19 pandemic.
	Rababa M [69]	2020	Original research (Cross-sectional study)	Jordan	Sample: 248 community-dwelling older adults (aged 60–75) Period: Unspecified Main results: The majority of participants were found to have low levels of religious coping and spiritual well-being and high levels of death anxiety. Females were found to have higher levels of religious coping and lower levels of death anxiety than men. Religious coping and spiritual well-being were found to be significant predictors of death anxiety in older adults.
	Suresh M [70]	2020	Original research (Cross-sectional study)	United States	Sample: 310 homebound older adults Period: May–July 2020 Main results: The more positive religious coping individuals used, the less likely they were to fall into the high suicide risk category.
	Chow SK [71]	2021	Original research (Cross-sectional study)	Malaysia	Sample: 200 HCWs Period: unspecified Main results: HCWs scored higher in positive religious coping than negative religious coping. Positive coping statistically significantly predicted a reduction in anxiety and log-transformed depression score.
	Sen HE [72]	2022	Original research (Longitudinal study)	Canada; United States; United Kingdom; Australia	Sample: 549 caregivers Period: May–November 2020. Main results: Religion and spiritual beliefs and practices were positively associated with coping and coping was inversely related to psychological distress.

Note: n.a.—not applicable; HCW—healthcare workers. References reported do not follow numerical order but are clustered for topics. Other topics that emerged were: concern regarding the possible role of fasting in influencing the severity of the SARS-CoV-2 infection [73,74,75]; how religious worship adapted to gathering restrictions (e.g., online ceremonies etc.) and how faithful perceived this shift [76,77,78,79].

## Data Availability

Data supporting the study are available in the references of the studies included in the review.
